# Caregiving Between Spouse and Adult Child Caregivers of Older Adults with Cognitive Impairment

**DOI:** 10.1093/geroni/igab046.2920

**Published:** 2021-12-17

**Authors:** Nicole Garcia, Anna Papazyan, Sarah Choi, Yeonsu Song

**Affiliations:** 1 UCLA, Los Angeles, California, United States; 2 VA Greater Los Angeles Healthcare System, California, Los Angeles, California, United States; 3 University of California, Los Angeles, Los Angeles, California, United States

## Abstract

Prior studies of caregiving characteristics by type of caregivers are inconsistent, particularly those who are spouses and adult children. This study examined caregiving characteristics between spouses and adult children of cognitively impaired older adults. We analyzed phone-screening data from an ongoing trial of a dyadic sleep intervention program for persons with dementia and their caregivers. Data included spouse caregivers (n=52) and adult child caregivers (n=24). Nearly all participants (95%) lived with their care recipients (91% with dementia). Types of caregiving activities were measured by activities of daily living [ADLs] and instrumental ADLs [IADLs] with their levels of intensity (0 [total independent] to 3 [total dependent]). Care recipients’ sleep was measured by the Neuropsychiatric Inventory-Nighttime Behavioral Subscale (8 items). Analyses included Pearson correlations and t-tests. Adult child caregivers helped their care recipients at significantly higher levels as indicated by their measure of dependence in dressing (1.46±1.22 vs. 0.87±1.16, p=0.044), continence (1.22±1.38 vs. 0.54±1.04, p=0.021), laundry (2.87±0.46 vs. 2.13±1.24, p=0.007), and transportation (3.00±0.00 vs. 2.63±0.79, respectively; p=0.031) than spouse caregivers. Adult child caregivers also reported their care recipients having more difficulty falling asleep (56% vs. 19%, respectively; p=0.004) and having more numbers of sleep problems than spouse caregivers (3.54±2.08 vs. 2.48±1.51, respectively; p=0.014). The findings suggest that adult child caregivers may involve higher levels of caregiving responsibilities during daytime and nighttime, compared to spouse caregivers. Further research needs to explore complimentary ways to involve spouse and adult child caregivers in the care of this vulnerable population.

